# Development of the catfish 250K SNP array for genome-wide association studies

**DOI:** 10.1186/1756-0500-7-135

**Published:** 2014-03-10

**Authors:** Shikai Liu, Luyang Sun, Yun Li, Fanyue Sun, Yanliang Jiang, Yu Zhang, Jiaren Zhang, Jianbin Feng, Ludmilla Kaltenboeck, Huseyin Kucuktas, Zhanjiang Liu

**Affiliations:** 1 The Fish Molecular Genetics and Biotechnology Laboratory, Aquatic Genomics Unit, School of Fisheries, Aquaculture and Aquatic Sciences, and Program of Cell and Molecular Biosciences, Auburn University, Auburn, AL 36849, USA

**Keywords:** Catfish, Fish, Genome, SNP array, GWAS, Genotyping

## Abstract

**Background:**

Quantitative traits, such as disease resistance, are most often controlled by a set of genes involving a complex array of regulation. The dissection of genetic basis of quantitative traits requires large numbers of genetic markers with good genome coverage. The application of next-generation sequencing technologies has allowed discovery of over eight million SNPs in catfish, but the challenge remains as to how to efficiently and economically use such SNP resources for genetic analysis.

**Results:**

In this work, we developed a catfish 250K SNP array using Affymetrix Axiom genotyping technology. The SNPs were obtained from multiple sources including gene-associated SNPs, anonymous genomic SNPs, and inter-specific SNPs. A set of 640K high-quality SNPs obtained following specific requirements of array design were submitted. A panel of 250,113 SNPs was finalized for inclusion on the array. The performance evaluated by genotyping individuals from wild populations and backcross families suggested the good utility of the catfish 250K SNP array.

**Conclusions:**

This is the first high-density SNP array for catfish. The array should be a valuable resource for genome-wide association studies (GWAS), fine QTL mapping, high-density linkage map construction, haplotype analysis, and whole genome-based selection.

## Background

Catfish is the most important aquaculture species in the United States. In recent years, catfish industry encounters great challenges including devastating diseases which cause the largest economic loss. Improved brood stocks with a high level of disease resistance are desperately needed. Quantitative traits, such as disease resistance, are most often controlled by a set of genes involving a complex array of regulation [1-3]. The dissection of genetic basis of these traits requires large numbers of genetic markers.

Single nucleotide polymorphisms (SNPs) are now the markers of choice because they are the most abundant genetic variations widely distributed in the genome, and are generally bi-allelic polymorphisms that are amenable to automated genotyping [4]. SNPs are efficient for genome-wide association studies (GWAS) because linkage disequilibrium can be detected with high-density SNPs when dealing with complex traits. Simultaneous analysis of thousands of SNPs have enabled genome-wide association studies for performance and production traits in chicken [5,6], pig [7,8], cattle [9-11], horse [12] and sheep [13,14]. However, such studies have not been possible with most aquaculture species including catfish due to the lack of genome-wide SNP markers and high-throughput SNP genotyping platforms.

Until recently genome-scale SNP identification was a great challenge for most non-model species. The next-generation sequencing technologies enabled efficient identification of SNPs from genomes of various organisms [15]. With the availability of a large number of SNPs, the challenge then is how to genotype these SNPs efficiently and economically.

A variety of SNP array platforms are available, of which Illumina iSelect HD Custom BeadChip (Illumina, San Diego, CA), the Sequenom MassArray (Sequenom, San Diego, CA) and Affymetrix GeneChip Custom Array (Affymetrix, Santa Clara, CA) are widely used. More recently, Affymetrix adopted the Axiom genotyping technology that allows development of customized arrays containing 1,500 to 2.6 million SNPs [16]. These platforms differ in their requirements for SNP marker numbers, sample size, cost and automation.

SNP arrays have been developed in several livestock species, including cattle [17], horse [18], pig [19], sheep [20], dog [21] and chicken [22]. For instance, the Illumina BovineSNP50 Beadchip featuring approximately 54,000 informative SNP probes was first developed for detecting variations in cattle breeds [17]. Two cattle SNP arrays with higher density were recently developed [23]. In dog, Illumina developed the CanineSNP20 BeadChip with 20K SNPs, and the recent CanineHD BeadChip containing over 170,000 SNPs [24]. A 60K chicken SNP array powered by Illumina iSelect BeadChip was designed to contain 57,636 SNPs [22]. Recently, a high density 600K chicken SNP array was developed with Affymetrix Axiom genotyping technology [25]. Apparently, there are no technology barriers for the development of high density SNP arrays, but the economic challenge is still tremendous. Even though the unit cost per genotype is currently extremely low, the total costs for the high density SNP arrays with high numbers of SNPs can still be beyond the economic powers of researchers working with species within small research communities.

High-density SNP arrays have not been developed for aquaculture species. Only low to medium density arrays were used in several aquaculture species. The Illumina GoldenGate Assay was used to evaluate 384 rainbow trout SNPs, resulting in a validation rate of 48% for the tested SNPs [26]. The GoldenGate Assay was also used to genotype 384 catfish EST-derived SNPs to assess the factors affecting SNP validation rates [27]. A custom Illumina iSelect SNP array containing approximately 6K SNP markers from Atlantic salmon was developed and used for linkage mapping and QTL analysis [28,29].

SNP resources required for the development of a high-density SNP array have been developed in catfish. Over two million gene-associated SNPs were identified in channel catfish and blue catfish, respectively, using next-generation sequencing. Of these putative gene-associated SNPs, approximately 400,000-500,000 were of high quality [30]. In a recent study, over eight million SNPs were identified in channel catfish by whole genome sequencing of one wild and four aquaculture populations [31]. With the availability of these SNP resources, we report here the development and performance evaluation of the 250K catfish SNP array using the Affymetrix Axiom genotyping technology.

## Results and discussion

### Selection of SNPs for the SNP array

One of the most important goals of the SNP array development is to have a good coverage of the genome. The first task was to select a subset of SNPs from all identified SNPs, gene-associated as well as anonymous SNPs. From a large pool of the previously identified SNPs [30,31], the following selection criteria were used for the initial selection of SNPs: 1) For gene-associated SNPs, at least one but no more than two SNPs per transcript contig were selected; 2) For anonymous SNPs, one SNP was selected for small contigs of less than 4 kb, but two SNPs were selected for contigs larger than 4 kb, with their spacing being the largest within the contig. In addition, a set of sequence features were also considered for the selection of the initial SNPs (see Methods).

The initial in house selection resulted in 641,489 SNPs that were submitted to Affymetrix for *in-silico* analysis to assess the predicted performance on Axiom arrays. Both forward and reverse probes of each SNP were assigned with p-convert values, which were derived from a random forest model to predict the probability of the SNP conversion on the array. The model considers factors including probe sequence, binding energy and the expected degree of non-specific hybridization to multiple genomic regions (personal communication with Affymetrix). SNP probes with high p-convert values are expected to convert on the SNP array with high probability.

**Figure 1:**
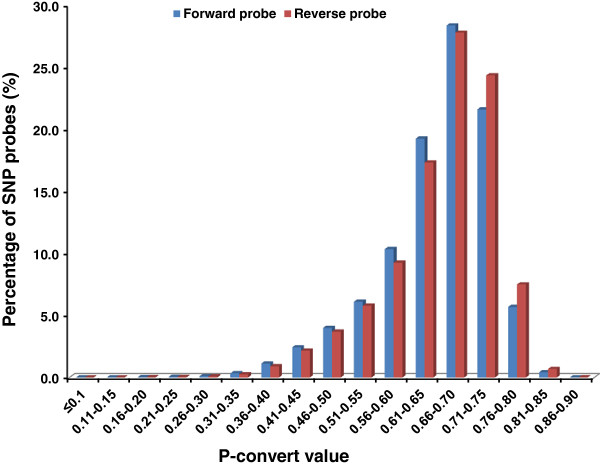
**Distribution of SNP probes based on p-convert values from Affymetrix *in-silico *** **analyses.**

A total of 495,671 SNPs passed the Affymetrix *in-silico* evaluation with various p-convert values, but the vast majority of SNPs had p-convert values greater than 0.5 (Figure 1). Because many more than needed SNPs passed the p-convert evaluation, only SNPs with p-convert values greater than 0.5 were further considered for inclusion on the array. For the SNPs with both probes passing the p-convert threshold, the probes with the higher p-covert values were selected. For the SNPs with both probes having relatively low p-convert values, both probes were selected to increase the conversion rate for the SNP. In the final SNP list, A/T and C/G SNPs were removed because such SNPs require twice the number of probes for genotyping.

### SNPs included on the 250K array

The SNPs used for the development of the catfish 250K SNP array are summarized in Table 1. A total of 250,113 SNPs were included in the 250K array including 103,185 (41.3%) gene-associated SNPs and 146,928 (58.7%) anonymous SNPs. Of the gene-associated SNPs, 32,188 were from SNPs identified from channel catfish, 31,392 were from SNPs identified from blue catfish, and 39,605 were inter-specific SNPs identified between channel catfish and blue catfish (Table 1, also referred to as inter-specific SNPs).

**Table 1:** Summary of SNPs used for the catfish 250K SNP array design

**SNP sources**	**SNPs selected for array**	**SNPs passed Affymetrix evaluation**	**SNPs included on the array**
Gene-associated SNPs			
Channel catfish	93,699	72,202	32,188 (12.9%)
Blue catfish	59,464	48,900	31,392 (12.6%)
Inter-specific	83,549	72,260	39,605 (15.8%)
Anonymous SNPs			
Channel catfish	404,777	302,309	146,928 (58.7%)
Total SNPs	641,489	495,671	250,113 (100%)

A total of 316,706 SNP probes were synthesized for the interrogation of these 250,113 SNPs, 66,593 SNPs of which were tiled with two probes (Table 2). In addition to SNP probes, 2,000 data quality control (QC) probes were included on the SNP array serving as negative controls. The QC probes were non-polymorphic 31-mers randomly picked from non-repetitive regions of catfish genome. We selected 1,000 QC probes with A or T at the 31st base, and another 1,000 QC probes with G or C at the 31st base.

**Table 2:** Summary of the catfish 250K SNP array

**SNP array**	**Number**
Total number of SNPs	250,113
Number of SNPs tiled with single probe	183,520
Number of SNPs tiled with two probes	66,593
Total number of probes	316,706
Number of data quality control probes	2,000

Inclusion of gene-associated SNPs should enhance the conversion rates because genes and their associated sequences are more unique in the genome than the non-coding genomic sequences. In addition, as genes are broadly distributed across all 29 pairs of chromosomes of the catfish genome [30], SNPs derived from genes should reflect the distribution of genes within the genome. However, as genes are not entirely evenly distributed, inter-marker spacing is not equal. Genomic SNPs from anonymous regions would fill the gaps. A subset of inter-specific SNPs are included, which are useful for genetic analysis of the inter-specific hybrid catfish system. The hybrid catfish produced by crossing channel catfish female with blue catfish male are widely used in the catfish industry because the hybrids possess several superior performance and production traits to both of their parents.

### Distribution of SNP spacing

**Figure 2:**
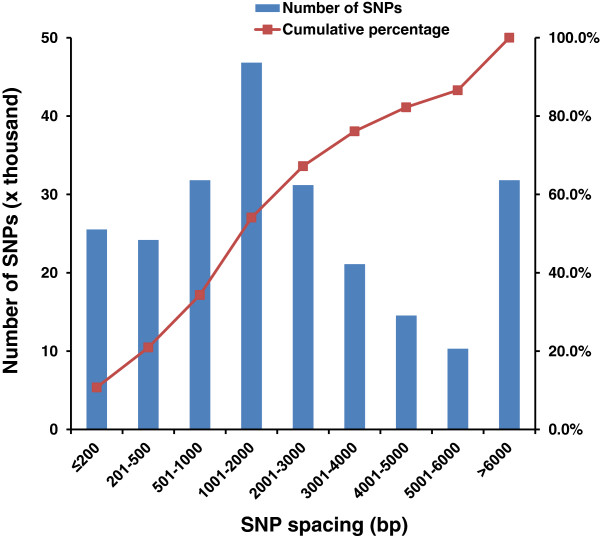
**Distribution of inter-SNP spacing of SNPs on the array.** SNP intervals were determined based on current catfish assembly.

We were unable to determine the absolute SNP coordinates and thereby their genome distribution because a fully assembled catfish genome is still not available. After the completion of SNP array development, a draft catfish genome assembly was generated (unpublished). To assess the SNP distribution, the inter-SNP spacing was evaluated using this draft genome assembly. A total of 248,308 SNPs (99.3%) with flanking sequences were uniquely mapped to 11,017 genome scaffolds which span a total of 785.6 Mb, approximately 80% of the genome. As shown in Figure 2, a total of 237,291 SNPs with inter-SNP spacing were examined. A total of 49,718 SNPs had a small inter-SNP spacing of less than 500 bp, and 31,811 SNPs had an inter-SNP spacing of 500–1000 bp. The largest number of SNPs (46,804) had an inter-SNP spacing of 1000–2000 bp. A total of 31,184 had a marker spacing of 2000–3000 bp, 21,100 had a marker spacing of 3000–4000 bp, 14,538 had a marker spacing of 4000–5000 bp, 10,316 had a marker spacing of 5000–6000 bp, and 31,820 had a marker spacing of more than 6000 bp. Cumulatively, 34.4% SNPs had a marker spacing of less than 1 kb; 52.2% SNPs had marker spacing of 1–6 kb; and 13.4% had a marker spacing of greater than 6 kb (Figure 2).

Due to the lack of a fully assembled genome sequence, the inter-marker spacing is probably underestimated. The current draft genome assembly had a total genome size of 830.5 Mb, but the catfish genome is approximately 950 Mb. In addition, the spacing from the most external SNPs of each scaffold to the next marker is not included in the assessment. Therefore, the overall marker spacing could have been slightly larger.

The SNP distribution was also evaluated with regard to association with bacterial artificial chromosome (BAC) end sequences (BES). A total of 22,051 SNPs on the array are associated with 16,832 BES derived from 14,849 BAC clones. Accordingly, such SNPs are associated with 2,853 (86.3% of 3,307) contigs from the catfish physical map developed by Xu et al. [32], including 1,141 contigs that were not able to integrate with linkage map constructed mainly using microsatellite markers [33]. Such BAC associated SNPs are useful because they are separated by a known distance in the genome (BAC insert size of 161 Kb on average [32]), and their use can facilitate full integration of genetic linkage and physical maps.

The catfish have a genome size of ~ 1 billion base pairs. With the 250,000 SNPs, theoretically, the average SNP marker intervals are about 4 kb in the catfish genome. We should acknowledge that it is too costly to develop a SNP array with millions of markers for catfish, like it has been done in human and other model species, since far less funding support is available for aquaculture species. For the same budget related reason, the number of samples genotyped for GWAS is limited for aquaculture species as well. Therefore, different strategies should be utilized when conducting genome-wide genetic analysis using the catfish 250K SNP array. The genome regions underlying production and performance traits can be first located through the whole genome scanning with the 250K SNPs genotyped from hundreds of samples. To further investigate the SNP effects, it’s cost-saving to use other SNP genotyping platforms such as Sequenom MassArray (Sequenom, San Diego, CA) to genotype thousands of individuals with denser SNPs from the targeted regions.

### Performance of the catfish 250K SNP array

#### Genotyping performance of the SNP array

Performance of the SNP array was examined by genotyping unrelated catfish samples from wild populations and catfish backcross families. As summarized in Table 3, a total of 204,437 SNPs (81.7%) were converted in wild catfish samples, of which 137,459 (55.0%) were polymorphic. The SNP conversion rate and polymorphic rate in BC1 catfish were relatively lower than those in unrelated wild catfish, as expected. However, higher conversion rate and polymorphic rates were observed in BC3 catfish than in BC1 catfish as well as in unrelated wild catfish (Table 3). The reason is that the BC3 catfish possess a higher fraction of “channel catfish” genome than BC1 catfish, as backcross families were produced by backcrossing hybrid catfish with channel catfish. Therefore, higher proportions of intra-specific SNPs from channel catfish were expected to convert in BC3 catfish than in BC1 catfish. Furthermore, the BC3 catfish possess hybrid genome regions, therefore, the inter-specific SNPs that only exist in the hybrids were expected to convert in BC3 rather than in wild catfish.

**Table 3:** Performance assessment of the catfish 250K SNP array

Samples*	**Samples processed**	**Samples passed**	SNPs converted**	**Polymorphic SNPs**	Avg. SNP call rate***
Wild catfish	192	182 (94.8%)	204,437 (81.7%)	137,459 (55.0%)	99.4%
BC1	192	179 (93.2%)	198,583 (79.4%)	130,685 (52.3%)	99.7%
BC3	192	192 (100%)	218,440 (87.3%)	156,357 (62.5%)	99.8%

*BC1 denotes the catfish from 1st generation of backcross, and BC3 denotes the catfish from 3rd generation of backcross. **SNPs on the array that work. ***The average percentage of samples whose genotypes were successfully measured for given converted SNPs.

Although polymorphism ultimately dictates how useful the array, the results present herein is related to the testing samples used in this study. In the case of the catfish 250K SNP array, the situation is further complicated by inclusion of intra-specific as well as inter-specific SNPs. Inter-specific SNPs are not expected to be polymorphic within channel catfish or blue catfish, but are polymorphic in inter-specific hybrids. One obvious question we are interested to ask is how many of the 250K SNPs represented real SNPs (the validation rate). Here, a total of 137,459 SNPs were polymorphic in wild fish; 130,685 SNPs were polymorphic in BC1 fish, and 156,357 SNPs were polymorphic in BC3 fish. Taken together, of the 241,812 converted SNPs, a total of 200,860 SNPs (83.1%) were polymorphic in at least one testing population, demonstrating a high SNP validation rate.

**Figure 3:**
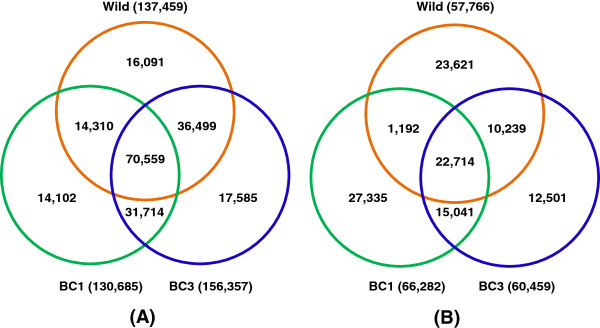
**Comparisons of polymorphic SNPs and monomorphic SNPs among three groups of fish. (A)** Polymorphic SNPs, **(B)** Monomorphic SNPs. Wild, unrelated wild channel catfish, BC1, 1st generation of backcross progeny, and BC3, 3rd generation of backcross progeny.

Comparisons of polymorphic and monomorphic SNPs among wild catfish, BC1 and BC3 catfish indicated that a large number of SNPs (70,559) were polymorphic in all three examined groups of fish (Figure 3A), while a relatively small number (22,714) of SNPs were not polymorphic in any of the three fish groups tested (Figure 3B). These 22,714 SNPs were most likely pseudo-SNPs, although they could still be real SNPs when more fish are tested.

The catfish hybrid system is not only important to the industry but also interesting for the genetic studies because the inter-specific hybrids exhibit significant heterosis. The high conversion and polymorphic rates achieved by genotyping catfish from backcross hybrid families as well as wild populations suggested good performance and utility of this SNP array.

#### Assessment of informativeness of SNPs on the array

**Figure 4:**
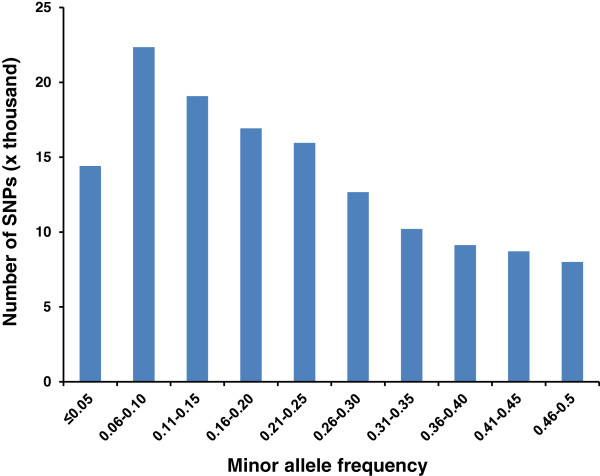
Distribution of minor allele frequencies.

Marker informativeness is reflected in minor allele frequencies (MAFs) as SNPs are most often bi-allelic markers. In order to assess the informativeness of the SNPs on the array, the minor allele frequencies of the SNPs were determined in wild population. The genotypes of 137,459 polymorphic SNPs in wild catfish samples were used for the analysis. Distribution of minor allele frequencies with intervals of 0.05 was shown in Figure 4. Overall, most polymorphic SNPs had a MAF of greater than 0.05, with 22,349 between 0.06-0.10, 19,084 between 0.11-0.15, 16,927 between 0.16-0.20, 15,961 between 0.21-0.25, 12,667 between 0.26-0.30, 10,207 between 0.31-0.35, 9,133 between 0.36-0.40, 8,710 between 0.41-0.45, and 8,006 between 0.46-0.50. Such distribution of the minor allele frequencies indicates that the array is likely very informative in most cases. Obviously, the higher MAF a SNP has, the more informative it will be. However, SNPs with low MAFs (rarer variants), possibly with larger effects, therefore, are essential in genome-wide association analysis as well [34].

#### Relationships between design score and SNP performance

**Figure 5:**
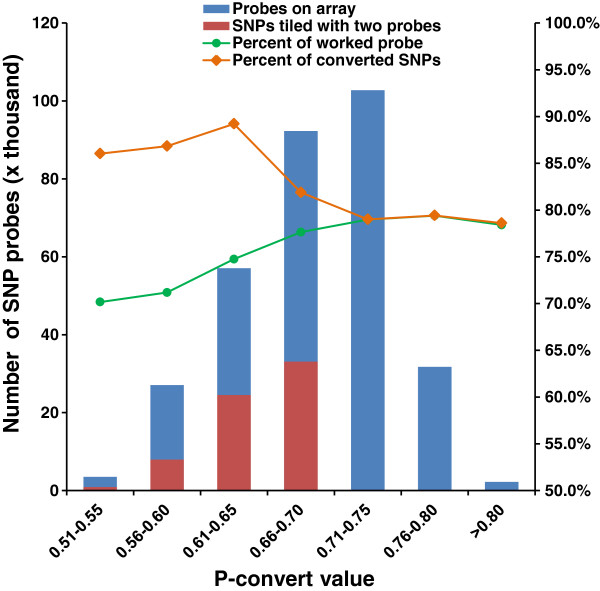
Relationships between Affymetrix design scores and SNP probe performance.

As the p-convert value is an important factor for the selection of SNPs, it is interesting to analyze its relationships with SNP performance. As shown in Figure 5, the p-convert values were positively correlated with the performance of the SNP probes, the higher the p-convert values were, the better performance of the probes was. However, once the p-convert values reached 0.7 or higher, further increase in p-convert values did not have additional effect on probe performance (Figure 5). This relationship holds not only for percent of probes that worked, but also for percent of converted SNPs. The spike in percent of converted SNPs with probes having lower p-convert values is an artifact due to the inclusion of two probes per SNP for SNPs with relatively lower p-convert values (Figure 5). Apparently, the p-convert value serves well as a parameter for the prediction of SNP probe performance.

#### Performance of gene-associated SNPs versus anonymous SNPs

**Figure 6:**
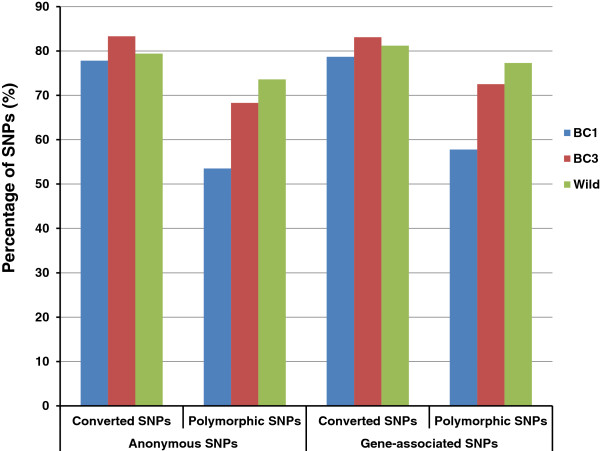
Performance between gene-associated SNPs and anonymous SNPs.

On the catfish 250K SNP array, 179,116 SNPs were identified from channel catfish, of which 146,928 were anonymous SNPs while 32,188 were gene-associated SNPs. To compare the performance of gene-associated SNPs and anonymous SNPs, the conversion rates and percentages of polymorphic SNPs were analyzed. As shown in Figure 6, there is no significant difference in performance between gene-associated SNPs and anonymous SNPs, with the conversion rates and polymorphic SNP percentages of gene-associated SNPs being slightly higher, by a couple of percentage, than those of anonymous SNPs in all three examined populations.

#### Performance of intra-specific and inter-specific SNPs

**Figure 7:**
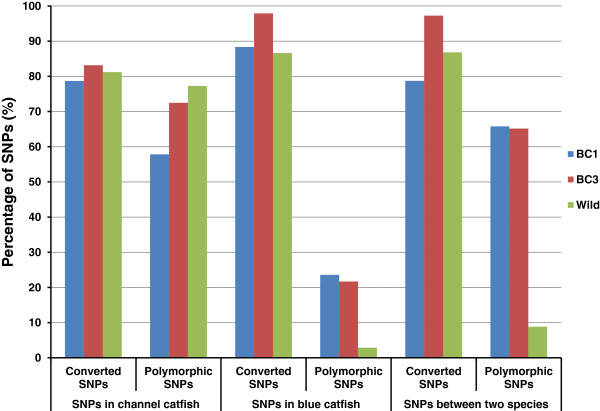
Performance between intra-specific SNPs and inter-specific SNPs.

The performances of 32,188 intra-specific SNPs in channel catfish, 31,392 intra-specific SNPs in blue catfish and 39,605 inter-specific SNPs between the two species were examined as shown in Figure 7. As expected, the highest percentage of polymorphic SNPs was converted from SNPs in channel catfish when being genotyped in channel catfish samples from the wild population. In contrast, the intra-specific SNPs identified from blue catfish had a very low polymorphic rate in wild channel catfish population. Similarly, only 8% inter-specific SNPs were polymorphic among wild channel catfish. However, such inter-specific SNPs performed really well in inter-specific backcross families, as expected (Figure 7).

#### Performance of transition and transversion SNPs

**Figure 8:**
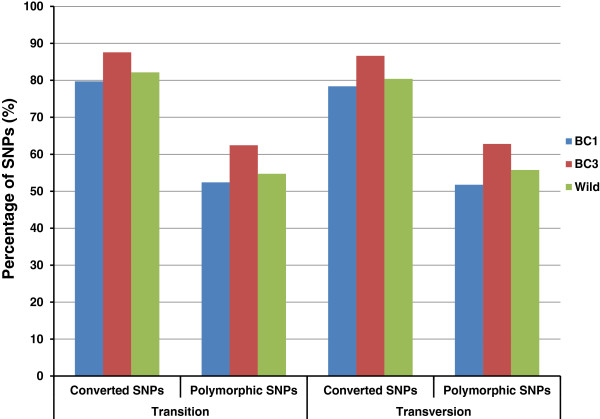
Performance between transition SNPs and transversion SNPs.

Of the 250,113 SNPs on the array, 75.9% are transitions and 24.1% are transversions. Transition SNPs are more abundant than transversion SNPs, with an estimated ratio of 1.8-1.9 in catfish among gene-associated SNPs [30,35]. The exclusion of A/T and G/C SNPs in the design stage of the SNP array reduced the fraction of transversion SNPs. It is interesting to examine the performance of these two types of SNPs. As shown in Figure 8, the two types of SNPs have nearly identical conversion rates and polymorphic rates, suggesting that they should not be different in their performance for genotyping.

### SNP transferability to other related catfish species

To assess the utility of the catfish 250K SNP array in the related catfish taxa, a set of DNA samples were tested from blue catfish (*Ictalurus furcatus*), brown bullhead catfish (*Ameiurus nebulosus*) and white catfish (*A. catus*). As summarized in Table 4, the overall SNP conversion rates across these species were actually quite high, with a minimal rate of 50.4% in brown bullhead and as high as 77.2% in D&B strain of blue catfish. However, the polymorphic rates were much lower, more than 10 times lower than the conversion rates in most cases. For instance, the polymorphic rates were 7.8% and 3.9% in Rio Grande and D&B strain of blue catfish, respectively, for all 250,113 SNPs on the array, as compared to 43.5% and 18.7% polymorphic rates for SNPs identified from blue catfish (31,392) when tested in the same samples. The SNPs on the array had low polymorphic rates for brown bullhead and white catfish as well, ranging from 4.4-5.1%. Taken together, these results suggested that the probes designed from channel catfish and blue catfish sequences could actually hybridize to the genomic DNA of brown bullhead and white catfish, but the bases at the SNP sites were not polymorphic in the two catfish species.

**Table 4:** Transferability of SNPs to other catfish species

**Species**	**Latin name**	Converted SNPs*	Polymorphic SNPs*	Converted SNPs**	Polymorphic SNPs**
Blue catfish (Rio Grande)	*Ictalurus furcatus*	190,867 (76.3%)	19,549 (7.8%)	25,722 (81.9%)	13,667 (43.5%)
Blue catfish (D&B)	*I. furcatus*	193,039 (77.2%)	9,684 (3.9%)	25,109 (80.0%)	5,859 (18.7%)
Brown bullhead catfish	*Ameiurus nebulosus*	126,076 (50.4%)	12,649 (5.1%)	17,739 (56.5%)	1,376 (4.4%)
White catfish	*A. catus*	129,716 (51.9%)	12,833 (5.1%)	18,286 (58.3%)	1,452 (4.6%)

*All 250,113 SNPs on the array, **SNPs from blue catfish (31,392).

In spite of the low polymorphic rates, the number of SNPs that were polymorphic was still notable with various species of catfishes. Over 12,000 SNPs were polymorphic for bullhead catfish and white catfish, suggesting its applicability for genetic analysis in related catfish taxa. The polymorphic rates evaluated here are probably underestimated because only 10 individuals were genotyped. Polymorphic rates would increase if more fish had been genotyped. Although these estimates are quite preliminary, the very low polymorphic rates observed in D&B strain of blue catfish suggest that this strain may have experienced inbreeding and might have had a small number of founders, at least twice less diverse than the Rio Grande strain (Table 4).

## Conclusions

In this study, we report the development of the catfish 250K SNP array using Affymetrix Axiom genotyping technology. The SNPs were well-spaced in the genome. Distribution of minor allele frequency indicated that SNPs with uniform MAFs were included on the array. The performance evaluation of the SNP array by genotyping samples from pedigree families and unrelated wild populations suggested high SNP conversion rates (~80%) and high polymorphic rates (over 50%) can be obtained in all the examined samples. Technically, we showed that the Affymetrix design score (p-convert value) adequately predicted SNP probe performance and the inclusion of alternative probes greatly increased the SNP conversion rates, especially for SNPs with probes that had low design scores. The catfish 250K SNP array should be valuable for genome-wide association studies, fine QTL mapping, high-density linkage mapping, haplotype analysis, and whole genome-based selection.

## Methods

### Ethics statement

All procedures involving the handling and treatment of fish used during this study were approved by the Auburn University Institutional Animal Care and Use Committee (AU-IACUC) prior to initiation.

### SNP selection and array design

Gene-associated SNPs were generated from Liu et al. [30]. Anonymous SNPs from non-coding genomic regions were from Sun et al. [31]. SNPs were filtered following the specific requirements for the Affymetrix SNP array design. Flanking sequences of 35 bp for each SNP were extracted where no other variations (SNPs and/or Indels) were allowed within 30 bp of SNPs. The balanced A/T/G/C of flanking sequences was required with GC content of 30%-70%. No repetitive elements were allowed in flanking sequences. In addition, single base repeats of G or C greater than 4 and A or T greater than 6 were not allowed.

All SNPs passing the in house selection using the above criteria were submitted to Affymetrix for design score assessment, where a p-convert value was assigned to each of the two probes flanking a SNP, respectively. SNPs with probes of high p-convert values were more likely to be convertible. A p-convert value threshold was determined by excluding the tail of lowest performing probes to facilitate selection of final SNP list. For the SNPs with both probes passing the p-convert value threshold, only the probe with the greater value was selected. For the SNPs with both probes having low p-convert values, both probes were selected to ensure a high probability of conversion.

To select well-spaced SNPs, at least one but no more than two SNPs per transcript contig were selected for gene-associated SNPs. At the time of the SNP selection, only the initial preliminary catfish genome assembly was available (255,858 contigs with mean length of 2,996 bp and N50 of 6,027 bp, unpublished data). The preliminary assembly was used to facilitate SNP selection according to contig length for the anonymous SNPs. One SNP per contig was selected from the contigs with lengths less than 4 kb. Two SNPs per contig were selected from the contigs with lengths greater than 4 kb. For the two SNPs selected from one contig, the SNPs with the largest distances were selected to ensure good spacing in the genome.

In addition, A/T and C/G SNPs were not selected because the two alleles of these SNPs match the same dye, and additional distinct probes in different physical locations on the array are required to distinguish them. Non-polymorphic 31-mers were randomly picked from non-repetitive regions of the genome for data quality control (QC) probes. The QC probes along with the final list of SNPs were submitted to Affymetrix for production of Axiom genotyping arrays.

### Assessment of SNP spacing

To assess the genome distribution of SNPs on the array, all the 250,113 SNPs with 35-bp up and downstream flanking sequences (71 bp in total) were aligned with the latest draft genome assembly now available (62,461 scaffolds with N50 of 3 Mb, covering a total size of 850 Mb, unpublished) using BLAST to determine SNP positions. The inter-SNP spacing was determined based on SNP positions in the scaffolds. The distance between SNPs at the end of sequences and the next SNP was not included because it’s not possible to assess their inter-marker interval. Similarly, SNP flanking sequences were aligned with the catfish BAC end sequences (BES) [36,37] to identify BES associated SNPs.

### SNP array performance evaluation

#### Fish sources

Three different sample sources were used for genotyping to assess the SNP array performance: 1) 192 unrelated channel catfish from wild populations; 2) 192 catfish hybrids from the 1st generation of backcrossing and 3) 192 catfish hybrids from the 3rd generation of backcrossing. Samples from wild populations were channel catfish collected for a previous study [38]. The hybrids from the 1st generation of backcrossing were produced by backcrossing the inter-specific F_1_ hybrids (channel female x blue male) with a male channel catfish, and the 3rd generation of backcross hybrids were produced by backcrossing the 2nd generation of backcross hybrids with a male channel catfish.

#### DNA isolation

Blood samples (300-500μl) were collected in a 1-ml syringe and immediately expelled into a 15-ml tube containing 5 ml of cell lysis buffer (100 mM NaCl, 10 mM Tris, pH 8, 25 mM EDTA, 0.5% SDS, and 0.1 mg/ml freshly made proteinase K) for DNA isolation. DNA was isolated as previously described [39,40]. Picogreen dye (Quant-iT Pico Green, Invitrogen) was used in order to quantify double-stranded DNA according to the manufacturer’s protocol. The integrity of DNA samples was checked by 1% agarose gel electrophoresis stained with ethidium bromide.

#### SNP genotyping

Genomic DNA samples were arranged in a 96-well microtiter plate, and normalized to a final concentration of 50 ng/μl with a final volume of 10 μl. Genotyping with the catfish 250K SNP array was outsourced to GeneSeek (Lincoln, NE, USA).

#### SNP analysis

**Figure 9:**
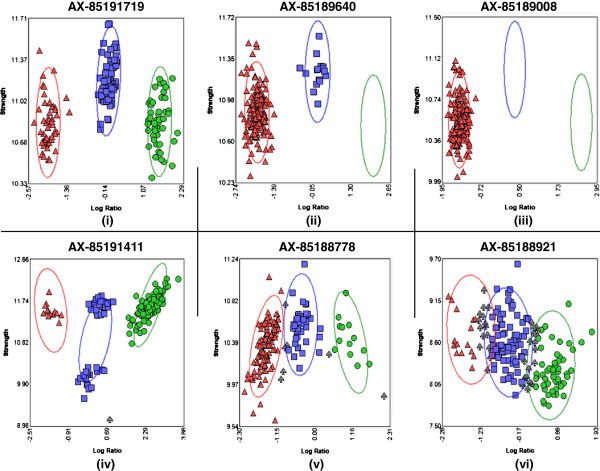
**Examples of six SNP/probeset categories.** SNPs/probesets were classified into six categories according to cluster properties: **(i)** “PolyHighResolution”; **(ii)** “NoMinorHom”; **(iii)** “MonoHighResolution; **(iv)**, “OTV” off-target variants; **(v)** “CallRateBelowThreshold”; and **(vi)** “Other” (see Methods).

Raw data in the form of CEL files were imported into the Affymetrix Genotyping Console software (v4.1) for quality control analysis and genotype calling using Axiom GT1 algorithm (Affymetrix). Samples with dish quality control (QC) value of 0.82 or better and call rate >0.97 were considered to have passed the quality control assessment. Following genotyping, SNPolisher (Affymetrix), an R package, was used to process the genotyping results. The package calculates the QC metrics for each SNP/probe set to determine its quality, and classify SNPs into six categories (Figure 9): (i) “PolyHighResolution” where three clusters are formed with good resolution; (ii) “NoMinorHom” where two clusters are formed with no samples of the minor homozygous genotypes; (iii) “MonoHighResolution” in which only one cluster is formed; (iv) “OTV”, off-target variants, where three good clusters are formed, but with one extra off-target cluster that is caused by sequence dissimilarity between probes and target genome regions [41]; (v) “CallRateBelowThreshold” where SNP call rate is below threshold, but other cluster properties are above threshold; and (vi) “Other” where one or more cluster properties are below threshold. The category (ii) was obtained when genotyping with related individuals such as in backcross families BC1 and BC3. In this study, SNPs of categories (i) to (iv) were regarded as convertible SNPs, and SNPs of categories (i) to (ii) were considered as polymorphic SNPs. The data used in this study are deposited in the National Animal Genome Research Program Aquaculture Genomics Data Repository (http://www.animalgenome.org/repository) and are also available upon request.

### SNP transferability to other related catfish species

A set of DNA samples were tested from other related catfish species, including blue catfish (*Ictalurus furcatus*) of Rio Grande strain (10) and D&B strain (10), 10 brown bullhead catfish (*Ameiurus nebulosus*) and 10 white catfish (*A. catus*). The DNA preparation, SNP genotyping and analysis were the same as mentioned above.

## Competing interests

The authors declare that they have no competing interests.

## Authors’ contributions

SL conducted the major part of the research including SNP selection, array design, data analysis, and manuscript preparation. LS, YL, FS, YJ, YZ, JZ and JF were involved in data analysis. MK and HK are involved in DNA sample preparation. ZL supervised the entire study and provided assistance for data analysis and manuscript preparation. All authors read and approved the final manuscript.
